# Dark Proteome Database: Studies on Disorder

**DOI:** 10.3390/ht9030015

**Published:** 2020-06-30

**Authors:** Nelson Perdigão, Pedro M. C. Pina, Cátia Rocha, João Manuel R. S. Tavares, Agostinho Rosa

**Affiliations:** 1 Instituto de Sistemas e Robótica, Instituto Superior Técnico, Universidade de Lisboa, 1049-001 Lisboa, Portugal; pedro.pina@laseeb.org (P.M.C.P.); catia.rocha@laseeb.org (C.R.); acrosa@isr.tecnico.ulisboa.pt (A.R.); 2 Instituto de Ciência e Inovação em Engenharia Mecânica e Engenharia Industrial, Departamento de Engenharia Mecânica, Faculdade de Engenharia, Universidade do Porto, 4200-465 Porto, Portugal; tavares@fe.up.pt

**Keywords:** proteins, prediction, intrinsic disorder

## Abstract

There is a misconception that intrinsic disorder in proteins is equivalent to darkness. The present study aims to establish, in the scope of the Swiss-Prot and Dark Proteome databases, the relationship between disorder and darkness. Three distinct predictors were used to calculate the disorder of Swiss-Prot proteins. The analysis of the results obtained with the used predictors and visualization paradigms resulted in the same conclusion that was reached before: disorder is mostly unrelated to darkness.

## 1. Introduction

With this work, we explored the difference between disorder and darkness (i.e., the distinction between intrinsically disordered proteins (IDP’s) [1] and their relationship with the dark proteome (DP) [2]). As defined, disordered regions are those with evidence of structural heterogeneity [3] where some become well-structured in particular contexts, and “dark” regions, as defined in 2015 [2], are those that do not match any Protein Data Bank (PDB) [4] entry, where some PDB entries, which are frequently obtained from electron microscopy (EM) or nuclear magnetic resonance (NMR) [5], are highly disordered. On the contrary, any partial sequence aligned to a PDB entry is classified as “non-dark” because some structural information is known. In our previous work [2], using the predictor IUPred [3], we concluded that the dark proteome is mostly not disordered. A predictor had to be used at that time since only 62 proteins (data from 2014) existed with “disordered” annotations from a total of 546,000 proteins of Swiss-Prot. In our subsequent work [6] (data from 2016), the same 62 proteins remained, but among a set of 550,116 Swiss-Prot proteins. The following hypothesis was formulated: If we had employed another predictor, would we have gotten a different result? One can consider that intrinsically disordered predictors are roughly divided into three categories: (i) predictors based on physicochemical properties; (ii) predictors based on machine learning classifiers; (iii) predictors based on a meta-approach which combines multiple predictors. However, this classification is not strict, since some of the predictors fall into more than one of these categories [7]. Among the categories listed, we are only interested in the latter, since they incorporate more knowledge from different sources. Yet, that led to another question: Can the nature of the methods used by a predictor influence the results? In other words, how does the relationship between disorder and darkness change by using different methods? Moreover, do common patterns exist if change occurs? Since this kind of predictor does not directly predict IDPs from its input, but instead combines several IDP prediction methods, which results in a final prediction by taking into account all the results obtained.

The choice of the used predictors was made based on our previous works on this topic [2,6,8,9], and on the nature of the methods or programs that they use. Since we wanted to perform a comparison of the results obtained by a predictor that uses a certain type of methods, another one that uses different methods from the first one and, lastly, a predictor that uses different methods from the previous two predictors. META-Disorder (MD) [10] was chosen, due to the usage of a support vector machine classifier for the prediction of disorder. MD was previously applied on data from 2014 [8,9], but in this work, MD was applied to data from 2016 in order to observe the prediction evolution of these two datasets. An updated version of IUPred (IUPred2A) [11] was also studied. In this case, the reasons for the predictor were similar: IUPred [3] was used in one of our previous works [2], and IUPred2A, such as MD, incorporates different methods to calculate disorder. VSL2 [12] was also used as a predictor in this category due to the fact that it incorporates several methods to calculate disorder based on neural networks. Finally, to visualize darkness versus disorder, Venn diagrams, the usual 2D Plots [2,8,9], and a Parallel Coordinates [13] viewer for the Dark Proteome Database (DPD) [6] was adapted for demonstration.

## 2. Materials and Methods

Dataset: The set of protein sequences selected for this work study is from the Swiss-Prot release of July 2016, together with the protein structures extracted from PDB on July 2016, including the predictions from Aquaria [14], Protein Model Portal [15], and Predict Protein [16], according to their versions of July 2016 [6]. The Swiss-Prot dataset was composed of 550,116 proteins divided into four kingdoms: 19,370 protein sequences from Archaea, 332,327 from Bacteria, 181,814 from Eukaryota, and 16,605 from Viruses [6]. However, to maintain a fair comparison with the previous results [2,8,9], in the presented 2D plots and parallel coordinates representations, the used number of proteins was reduced to 18,999 in Archaea, 326,945 in Bacteria, 176,646 in Eukaryota, and 16,316 in Viruses.

Mapping Darkness: For each Swiss-Prot protein, each residue was categorized as “non-dark” if it met either one of the following criteria: if the residue was aligned onto the “ATOM” record of any PDB entry [4] in the corresponding Aquaria [14] matching structures entry—criterion A; if the residue was aligned onto a PDB entry in the corresponding UniProt entry—criterion B. All other residues were categorized as “dark”. We then calculated a “darkness” score (*D*) as defined in [2] and shown in Equation (1). Accordingly, if *D* = 0, it is PDB or a white protein, otherwise, if *D* = 1, it is a dark protein. Finally, if 0 < *D* < 1, it is a grey protein containing dark regions [2].

(1) $$D=\;\frac{number\;of\;dark\;residues}{total\;number\;of\;residues}$$

Mapping Disorder: For each Swiss-Prot protein, we estimated the intrinsic disorder using three prediction methods: MD, IUPred2A, and VSL2.

The MD method is a combination of four prediction methods: DISOPRED2 [17] a Support Vector Machine (SVM) based prediction of missing coordinates in X-ray structures; IUPred [3] for prediction of unstructured regions based on pairwise statistical potential; NORSnet [18] for prediction of unstructured loops; and Ucon [19] which is a specific contact-based prediction method. All of the four methods are optimized to predict residues missing from PDB structures under different circumstances. MD returns a score between 0 (zero) and 1 (one) for each amino acid, corresponding to the probability of the given residue being part of a disordered region.

The IUPred2A identifies Intrinsically Disordered Protein Regions (IDPRs) (i.e., regions that lack a stable monomeric structure under native conditions, based on a biophysics-based model). In other words, IUPred2A assumes that disordered regions are formed based on amino acids with less energy to form contacts. Therefore, for any protein sequence, as with MD, IUPred2A returns a score between 0 (zero) and 1 (one) for each residue. Currently, the IUPred2A algorithm is able to detect this context-dependent disorder in the case where the environmental factors are either a change in the redox state or the presence of an ordered binding partner. IUPred2A supersedes the previous IUPred algorithm, according to the authors, since it incorporates several resources, such as MobiDBlite [20], MobiDB 3.0 [21], and InterPro [22]. In this work, its long version was used [11].

The last predictor used was the VSL2 predictor, which is a combination of neural network predictors for both short (less than 30 residues) and long disordered regions. Each individual predictor is trained using the dataset containing sequences of that specific length. The final prediction is a weighted average, determined by a second layer predictor [12]. VSL2 applies not only the sequence profile, but also the result of sequence alignments from PSI-BLAST [23] and the secondary structure prediction from PHD [24] and PSIPRED [25]. As with the previous two predictors, VSL2 returns a score between 0 (zero) and 1 (one) for each amino acid, indicating the probability level of it being part of a disordered region. Here, the overall disorder for each protein is calculated as the sum of the disorder probability of each amino acid considered to be disordered (cut-off above 0.5), divided by the protein’s length.

Mapping Compositional Bias: For each Swiss-Prot protein, a compositional bias score was calculated by pooling all of the residues annotated as compositionally biased in the “Features” section of the corresponding UniProt entry—this number was then divided by the total number of amino acids [2].

Mapping Transmembrane: A transmembrane score was calculated for each protein by pooling all residues annotated as either intra- or transmembrane in the “Features” section of the corresponding UniProt entry—the calculated score was then divided by the protein’s length. Most of these UniProt annotations derive from machine-learning methods (www.uniprot.org/help/transmem) that are believed to predict transmembrane regions with more than 95% accuracy [26]. However, as stated before [2], a second set of transmembrane values obtained by running systematic predictions for all Swiss-Prot sequences with PROF [26] and PROFTMB [27] were used instead, which predict transmembrane helices and *β*-barrels, extremely similar.

**Figure 1:**
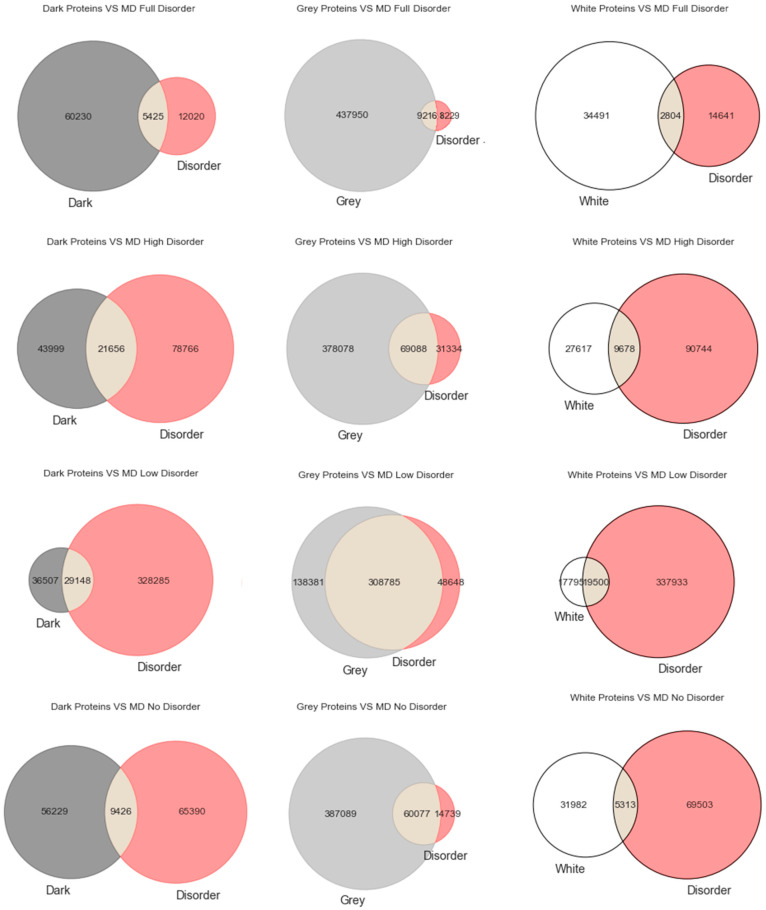
Venn diagrams built for the MD predictor as to darkness versus disorder.

**Figure 2:**
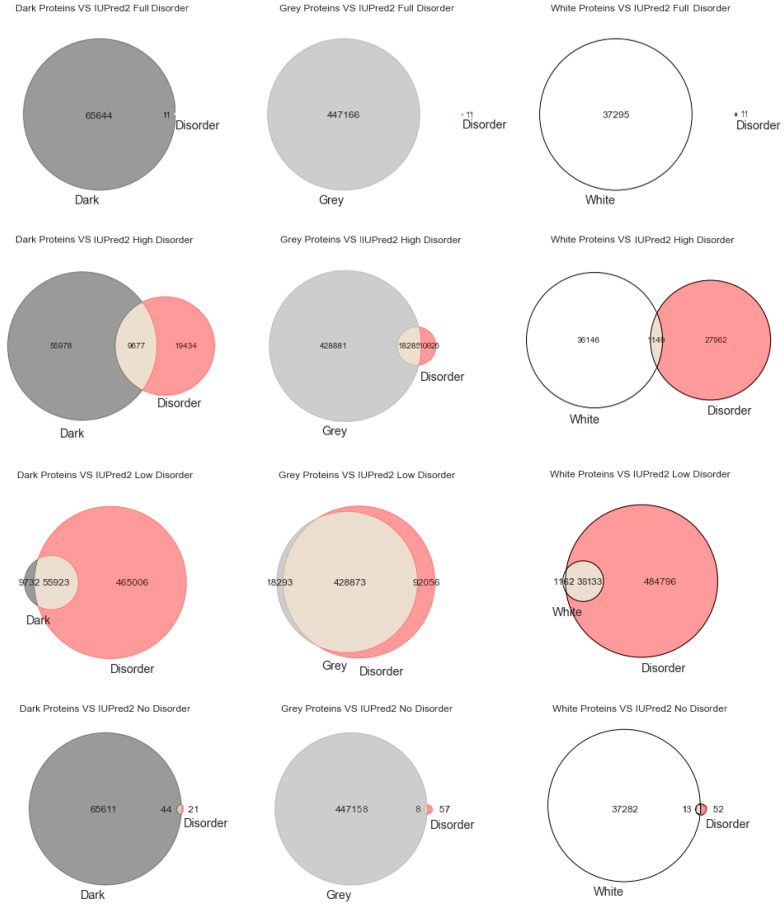
Venn diagrams built for the IUPred2A predictor as to darkness versus disorder.

**Figure 3:**
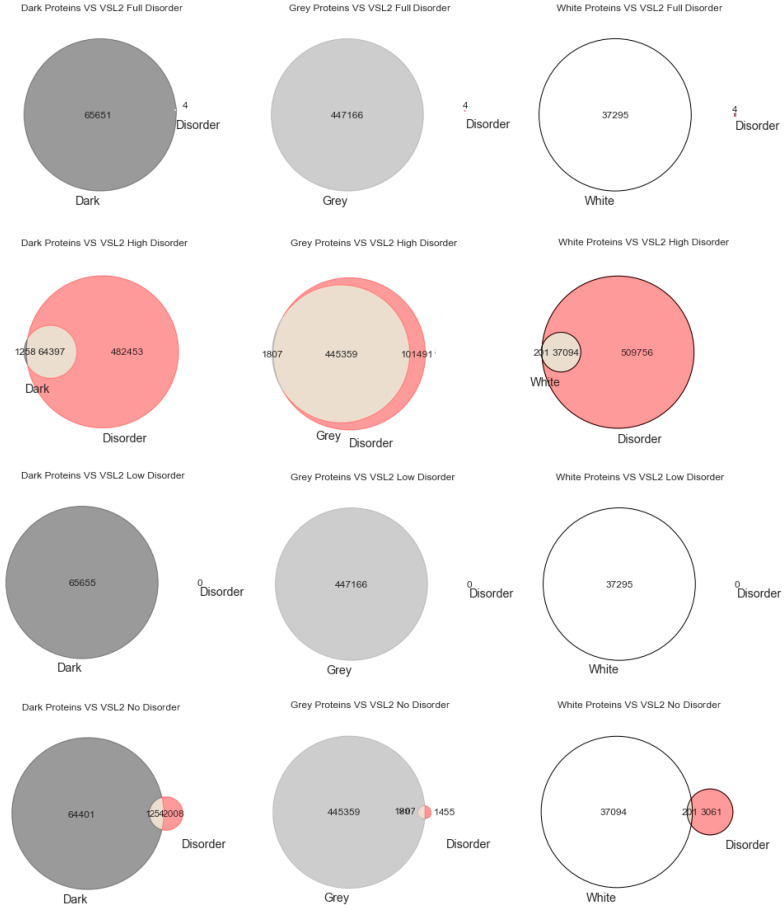
Venn diagrams built for the VSL2 predictor as to darkness versus disorder.

Venn Diagrams: Commonly also named primary diagrams, set diagrams, or logic diagrams, they are diagrams that show all possible logical relations between a finite collection of different sets. These diagrams depict elements as points in the plane and sets as regions inside closed curves. A Venn diagram consists of multiple overlapping closed curves, usually circles, each representing a set. The points inside a curve labelled *S* represent elements of set *S*, while points outside the boundary represent elements not in set *S*. This leads to easily readable visualizations, as shown in Figures 1–3.

**Figure 4:**
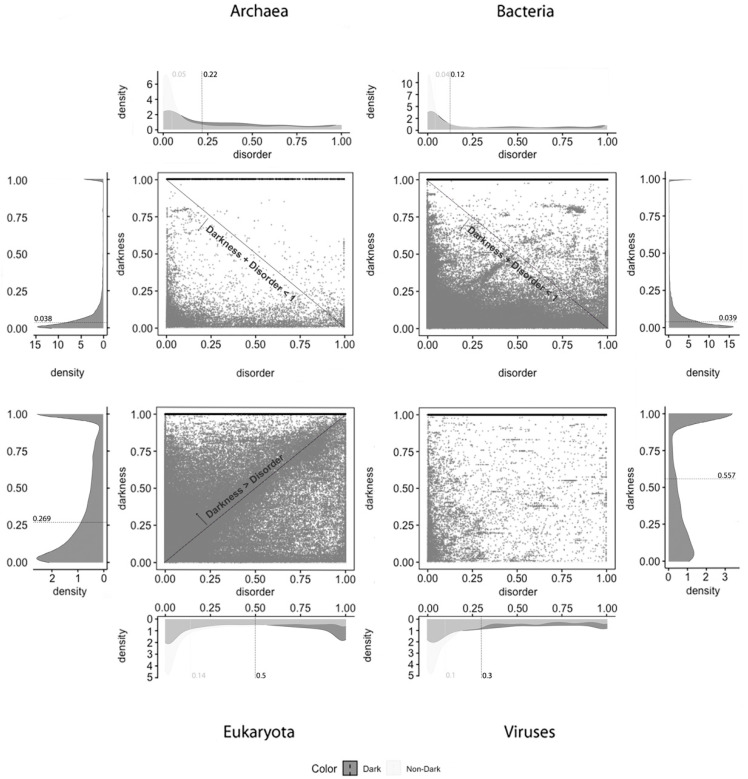
Darkness versus disorder 2D plot for the MD predictor.

**Figure 5:**
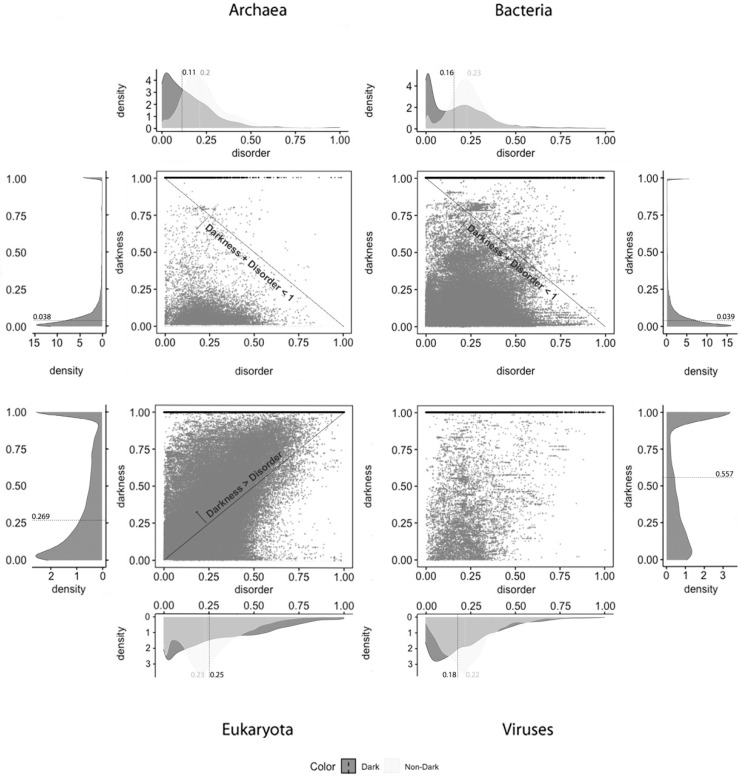
Darkness versus disorder 2D plot for the IUPred2A predictor.

**Figure 6:**
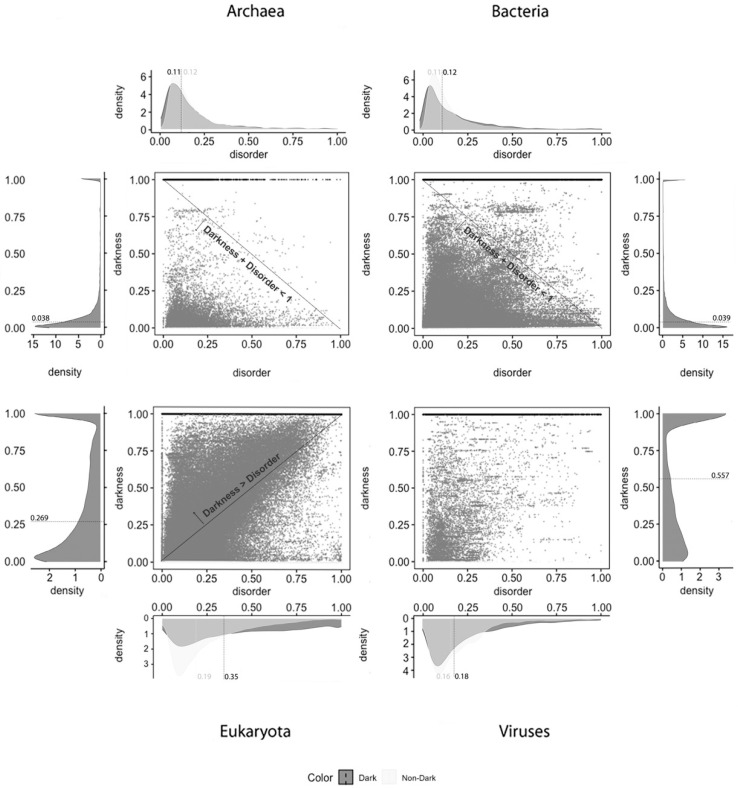
Darkness versus disorder 2D plot for the VSL2 predictor.

Density plots: The density plots in Figures 4–6 were built using Gaussian kernel density estimations [28], as implemented in the *stat_density* and *stat_density2d* functions of the *ggplot2* package in R with all parameters by default. In all of the density plots presented, the density values (*y* axis) are scaled so that the total area under the curve is equal to 1 (one). As a result, the density values depend on the range of values on the x axis, as shown in Figures 4–6. 

Two-Dimensional Plots: The *ggplot2* and *ggarrange* functions in R were used with default parameters and without manual editing, as shown in Figures 4–6.

Parallel Coordinates: Parallel Coordinates [13] is a method used to visualize multi-dimensional data, where each attribute is represented as a vertical line. These vertical lines are recommended to be uniformly spaced, where a line segment is then constructed by connecting the values on the vertical lines that correspond to the values of those attributes. A point in a *n*-dimensional space is represented as a polyline with vertices on the parallel axes—the position of the vertex on the *i*-th axis corresponds to the *i*-th coordinate of the point. Its main advantage is the simultaneous visualization of multi-dimensional data, as multiple attributes become potentially useful to facilitate exploring and pattern finding in the data under analysis, where theoretically there is no limit on data dimensionality. Parallel Coordinates were implemented in the Dark Proteome Database [6] using *node.js* and *d3.js*.

## 3. Results

Intrinsically disordered regions were believed to account for much of the dark proteome, especially in eukaryotes [29]. To explore that hypothesis, we start this section with the most classic paradigm of visualization of the three used in this work (i.e., Venn diagrams), in order to understand at first glance if darkness overlaps disorder or not. By counting cardinalities and percentages, a total of 550,116 Swiss-Prot proteins were obtained: dark proteins, 65,655 (12%); grey proteins, 447,166 (81%); and white/PDB proteins, 37,295 (7%).

As already mentioned, we used three predictors (MD, IUPred2A, and VSL2) to calculate disorder for each one of the 550,116 proteins. The protein disorder is a value between 0 (zero) and 1 (one) inclusive. We divided this disorder interval into four ranges: “Full Disorder”, when a protein is fully disordered in all its extension (i.e., protein disorder = 1, for every amino acid in the sequence); “High Disorder”, when a protein has some disorder in at least one or more disordered regions (i.e., 0.5 ≤ protein disorder < 1); “Low Disorder”, when amino acids have low disorder (i.e., protein 0 < disorder < 0.5); “No Disorder”, when no single amino acid is disordered in the protein (i.e., protein disorder = 0).

Starting with the MD predictor, 17,445 (3%) were found to be full disorder proteins, 100,422 (18%) high disorder proteins, 357,433 (65%) proteins with low disorder, and 74,816 (14%) with no disorder. Considering the IUPred2A, we obtained 11 (~0%) full disorder proteins, 29,111 (~5%) high disorder proteins, 520,929 (95%) proteins with low disorder, and 65 (~0%) proteins with no disorder. Finally, for the VSL2, the following predictions were obtained: 4 (~0%) full disorder proteins, 546,850 (99.4%) high disorder proteins, 0 (0%) proteins with low disorder, and 3262 (0.6%) proteins with no disorder. Therefore, every protein was classified in terms to darkness and disorder using the three predictors so that, with a minimum potential for bias, one can answer questions such as, what fraction of the proteins in the dark proteome is predicted to be disordered by each predictor?

Figure 1 displays the analysis made for the first predictor (MD), where the first row has only full disordered proteins (=1), the second row has proteins with levels of disorder lower than 1 (one) and higher than or equal to 0.5, the third row has proteins with levels of disorder lower than 0.5 and higher than 0 (zero), and finally the last row has only proteins with no disorder (=0). Observing Figure 1, one can conclude that darkness is not fully related to disorder, at least in what concerns dark proteins, as shown in Figure 1 in the left column, where this would be more expected under a classical point of view. As for the white/PDB proteins, as shown in Figure 1 in the right column, it would also be expected that no disorder exists, but one can still observe regions of contact in the Venn diagrams similar to dark proteins in terms of order of magnitude. The Venn diagram in the middle column of Figure 1 is where darkness and disorder mostly overlap. The corresponding 2D plots were generated, as shown in Figure 4, to detail and better understand these overlaps in all their extensions, not only for grey proteins.

Figure 2 depicts the analysis made for the second predictor (IUPred2A), where the first row has only full disordered proteins (=1), the second row has proteins with levels of disorder lower than 1 (one) and higher than or equal to 0.5, the third row has proteins with levels of disorder lower than 0.5 and higher than 0 (zero), and finally the last row has only proteins with no disorder (=0). The observations and conclusions that can be drawn from Figure 2 are similar to the ones from Figure 1. Again, the Venn diagrams in the middle column of Figure 2 is where darkness and disorder overlap more. The corresponding 2D plots were generated, as shown in Figure 5, to detail and better understand these overlaps, again, in all their extensions.

Lastly, Figure 3 shows the analysis for the third predictor (VSL2), where the first row has only full disordered proteins (=1), the second row has proteins with levels of disorder lower than 1 (one) and higher than or equal to 0.5, the third row has proteins with levels of disorder lower than 0.5 and higher than 0 (zero), and finally the last row has only proteins with no disorder (=0). The observations and conclusions that can be reached from this figure are similar to the ones made for Figure 1 and Figure 2. Again, the Venn diagrams in the middle column of Figure 3 is where darkness and disorder mostly overlap. For the same reason as in the previous two cases, the corresponding 2D plots were generated, as shown in Figure 6.

From what has been described, we can suspect that darkness is not explained by disorder. To clarify, we applied 2D plots to make a deeper analysis possible—the 2D scatter plots adopted in this work are the second paradigm of visualization. The visualization of the disorder and darkness scores on a 2D scatter plot makes it possible to discover a relationship (if any) between darkness and disorder, as was done before [2].

**Dark Proteome is Mostly Not Disordered.** We start this analysis focused on darkness, since it is transversal to all the predictors under study. From the analysis made, it can be globally observed by comparing the medians that occurred a slight reduction between the data of 2014 [2] and of 2016 [6,9] for all the kingdoms of life. Archaea, from 4 to 3.8%; Bacteria, from 4 to 3.9%; Eukaryota, from 28 to 26.9%; Viruses, from 65 to 55.7%. This reduction is likely due to the increasing quantity and/or quality of annotations in the dark regions of proteins already present at Swiss-Prot, among other factors, as shown in Table 1.

Concerning disorder predictors, and starting with the MD predictor, as shown in Figure 4, it is observed that dark proteins have mostly low disorder in Archaea (22%) and Bacteria (12%), getting medium values for disorder in Eukaryota (50%) and Viruses (30%), using the data of 2016. These values are a little higher than the ones obtained for the data of 2014 with the same predictor [10]: Archaea, 16%; Bacteria, 8%; Eukaryota, 40%; Viruses, 26%. Regarding non-dark proteins, their medians of disorder are lower than those for the dark proteins: Archaea, 5%; Bacteria, 4%; Eukaryota, 14%; Viruses, 10%. This result is in line with those published in 2019 [9]: Archaea, 5%; Bacteria, 4%; Eukaryota, 13%; Viruses, 8%. This predictor forecast that dark proteins have higher medians of disorder in all the kingdoms of life than the non-dark ones, as shown in Table 2. 

Applying the IUPred2A predictor, as shown in Figure 5, we observe that dark proteins mostly have low disorder in Archaea (11%), Bacteria (16%), Eukaryota (25%), and Viruses (18%), concerning the data of 2016. In comparison, these values are higher than those obtained from the data of 2014 [2] using the IUPred [3] predictor: Archaea, 0%; Bacteria, 0%; Eukaryota; 10%; Viruses, 3%. In respect to non-dark proteins, we obtained: Archaea, 20%; Bacteria, 23%; Eukaryota, 23%; Viruses, 22%. This resulted in higher values, but they are in line with those published in 2015 [2] where non-dark proteins presented higher disorder in comparison with the dark ones in terms of medians, except in Eukaryota, as shown in Table 3. 

The last analyzed predictor was the VSL2, as shown in Figure 6, for which we can conclude that dark proteins mostly have low disorder in Archaea (11%) and Bacteria (12%), getting medium values for disorder in Eukaryota (35%) and, again, lower values for Viruses (18%). Concerning non-dark proteins, the disorder values are similar to those of the dark proteins but lower, except for Archaea: Archaea, 12%; Bacteria, 11%; Eukaryota, 19%; Viruses, 16%. This is also a surprising result because the levels of disorder are very similar between dark and non-dark, where they are more or less similar, except in Eukaryota, as shown in Table 4. 

Concerning disorder standard deviation MD was the predictor that led to the widest disorder standard deviation, where the IUPred2A predictor obtained the lowest. The results for the VSL2 predictor stay between the results obtained by the other two predictors as shown in Table 5.

Finally, and regardless of the chosen predictor or the domain of life studied, we can observe that darkness is always greater than disorder for almost all proteins, since they are mostly above the diagonal darkness > disorder for Eukaryota. Concerning Archaea and Bacteria, we observe that most of the proteins occur in the region where darkness + disorder < 1, implying that dark and disordered regions were mostly disjointed. Finally, for Viruses, the 2D plot of disorder versus darkness is distinctly different from Archaea, Bacteria, and Eukaryota cases. The almost random distribution implies that darkness had almost no relationship to disorder in Viruses, however, it can still be observed that most of the proteins occur in the region where darkness + disorder < 1.

**Figure 7:**
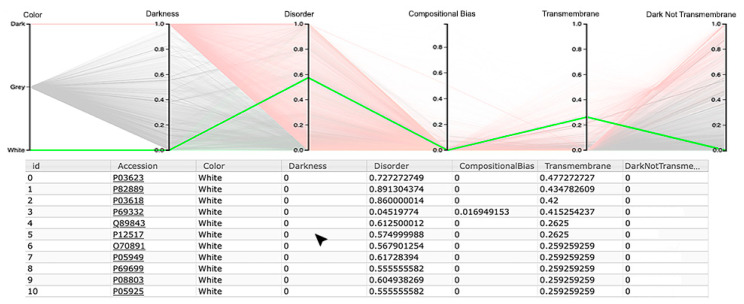
Parallel Coordinates Visualization System.

The third visualization paradigm adopted in this work was parallel coordinates [13], which is a method used to visualize multi-dimensional data with each attribute represented as a vertical line, where a visualization system as made available at the Dark Proteome Database (DPD) site (http://www.darkproteome.ws:8030/pc) was adapted. In this implementation, each plotline represents a protein attribute, such as darkness, disorder, compositional bias, transmembrane, and “dark residues not being transmembrane”, in terms of density values. Like it is adopted in parallel coordinates views, this visualization system permits the user to select a polyline representing a protein and brush it with a user-defined color. It is also possible to select certain lines, which will automatically hide the others, allowing the user to explore the correlation of attributes for a protein or a group of proteins in the canvas more easily. In addition, knowing that parallel coordinates views can suffer from over-plotting, the transparency of solid polylines through an alpha value is allowed. Putting all these features together, one can select proteins that exhibit certain attributes, allowing an individual protein study, if desirable, as shown in Figure 7 (i.e., this allows a protein or a group of proteins to be selected and directly highlighted via brushing for analysis). Finally, this visualization system allows the order of axes to be changed dynamically since it is very important for data patterns determination, together with column sorting. 

**Figure 8:**
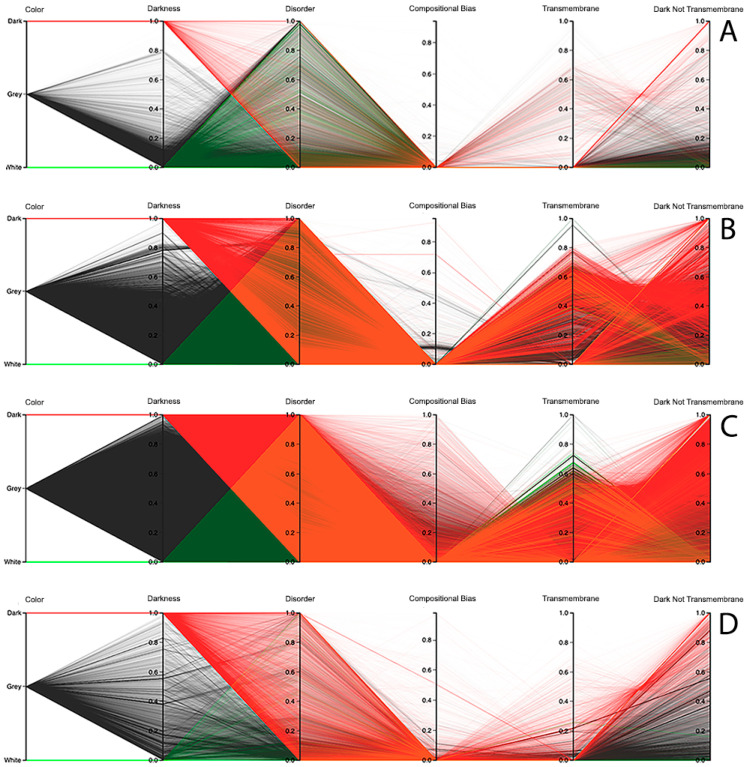
Darkness versus disorder representation using Parallel Coordinates for the MD predictor: (**A**) Archaea, (**B**) Bacteria, (**C**) Eukaryota, and (**D**) Viruses.

**Figure 9:**
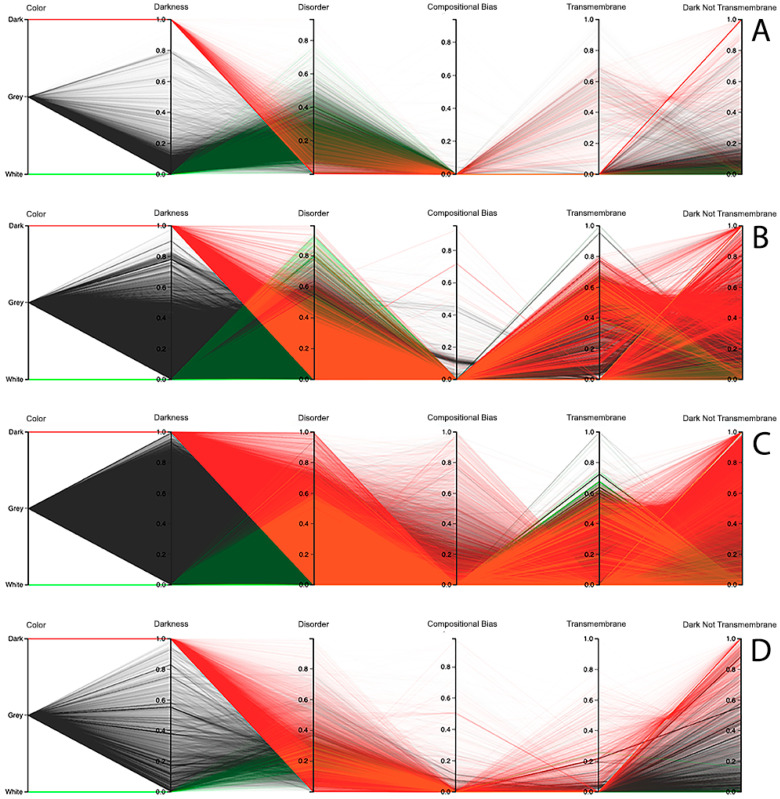
Darkness versus disorder representation using Parallel Coordinates for the IUPred2A predictor: (**A**) Archaea, (**B**) Bacteria, (**C**) Eukaryota, and (**D**) Viruses.

**Figure 10:**
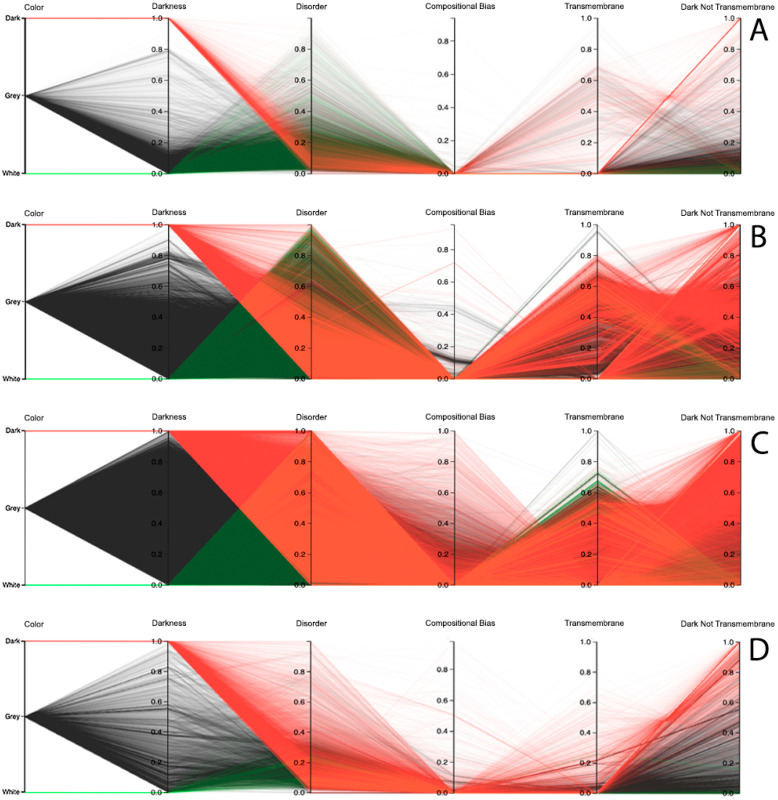
Darkness versus disorder representation using Parallel Coordinates for the VSL2 predictor: (**A**) Archaea, (**B**) Bacteria, (**C**) Eukaryota, and (**D**) Viruses.

Observing the results obtained with this third visualization paradigm for the MD, as shown in Figure 8, IUPred2A, as shown in Figure 9, and VSL2 predictors, as shown in Figure 10, we can conclude that darkness is poorly related with disorder for all the predictors in all domains of life, except for Eukaryota, especially when using the MD predictor, as shown in Figure 8C. All the other parallel coordinates representations visually reinforce what we had observed before—that the dark proteome is mostly not disordered, and that, sometimes, even the non-dark proteins have similar or even more disorder than the dark ones, depending on the used predictor.

Finally, information about compositional bias and transmembrane information was added to the parallel coordinates visualization system and, as had already happened, one could observe through the generated patterns that darkness is also poorly related to them, as shown in Figure 8, Figure 9 and Figure 10. 

**Dark Proteome is Mostly Not Transmembrane.** Transmembrane regions are also known as a contributor to darkness. To explore this assumption, for each protein we calculated the percentage of transmembrane residues [6]. Viewing these transmembrane and darkness scores on parallel coordinates, we observed that most dark proteins had no transmembrane residues (Dark Not Transmembrane axis), and we also see that a surprisingly large fraction of transmembrane residues were not dark, but they were mainly from white and grey proteins, especially in Bacteria and Eukaryota, as shown in Figure 8, Figure 9 and Figure 10. As a matter of fact, the last axis reinforces that very few dark amino acids are transmembrane, as shown in Figure 8, Figure 9 and Figure 10, with a few exceptions in Bacteria, as shown in Figure 8B, Figure 9B, and Figure 10B, and in Eukaryota, as shown in Figure 8C, Figure 9C, and Figure 10C, where the density values are slightly lower (meaning that some of dark amino acids are transmembrane) in comparison with the remaining cases.

**Dark Proteome is Mostly Not Compositionally Biased.** It is assumed that compositional bias was responsible for darkness. To explore this concept, for each protein we calculated the percentage of compositionally biased residues [6]. Viewing these compositional bias and darkness scores on parallel coordinates, we see, again, that darkness was greater than compositional bias for almost all proteins, as shown in Figure 8, Figure 9 and Figure 10, implying that, as expected, most compositionally biased residues were dark, this also being clear from the parallel coordinates, which indicate that most dark residues were not compositionally biased and that most dark proteins had very low compositional bias, even if marginally higher in Eukaryota, as shown in Figure 8C, Figure 9C, and Figure 10C.

**Figure 11:**
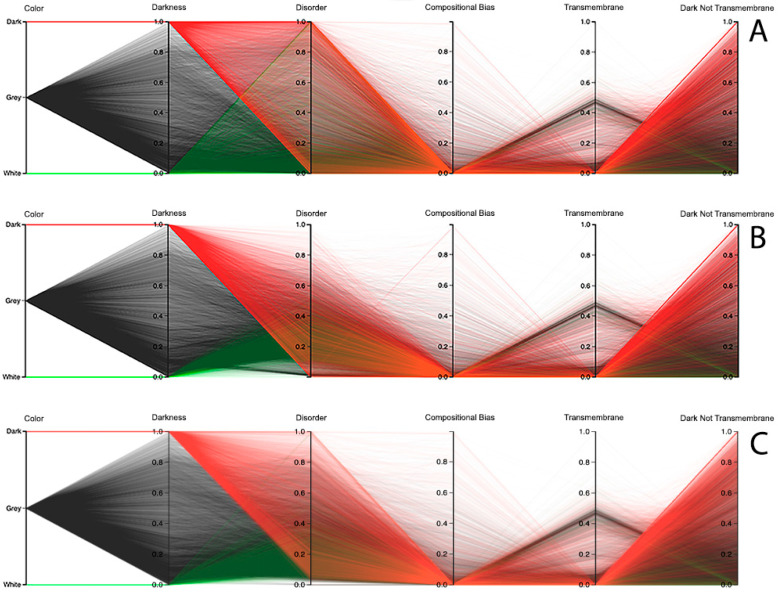
Darkness versus disorder representation using Parallel Coordinates in humans for the: (**A**) MD, (**B**) IUPred2A, and (**C**) VSL2 predictors.

We end this section by discussing the results for the human organism, where we also used the parallel coordinates paradigm, not only to analyze the relationship between darkness and disorder, but also the darkness relation with compositional bias and transmembrane residues. Starting with the disorder analysis, we observed that the results depend on the chosen predictor. For instance, looking at the results of the MD predictor, it can be observed that it has a small group of dark proteins fully disordered, as shown in Figure 11A. From there, the remaining dark proteins crossed almost all values in the disorder axis till they reached no disorder values. Curiously, the use of this predictor in white and grey proteins reached values of full disorder until the value of no disorder was reached, as shown in Figure 11A. Concerning the IUPred2A predictor, no dark proteins reached the value of full disorder, while a considerable group of them were considered highly disordered. It was also observed that the majority of white and grey proteins were classified as having low disorder, as shown in Figure 11B. The VSL2 predictor led to a pattern similar to the one obtained by MD, revealing a smaller group of dark proteins that were fully disordered. As in the case of the MD predictor, the remaining dark proteins crossed almost all values in the disorder axis; however, they were denser. Concerning the white and grey proteins, one can observe that their majority were classified as having low disorder; however, at least once the value of full disorder was reached, in addition to some sporadic high disorder cases, as shown in Figure 11C. Finally, it was verified that dark, grey, and white proteins have low compositional bias and few transmembrane residues, especially in dark and grey proteins, as shown in Figure 11. It can be noted that the values and polylines are the same for compositional bias, transmembrane, and dark not-transmembrane axis for all three predictors, as shown in Figure 11A–C.

## 4. Conclusions

In general, it can be stated that darkness is poorly related with disorder, based on the demonstration using the three studied predictors and adopted visualization paradigms. However, most importantly, and to answer the questions made in the introduction, something is clear: from the obtained results, it is possible to verify that the overall pattern was maintained, and that the different methods used by each predictor only affected the scattered level of the pattern. Since we are dealing with predictors and do not know which is the golden standard, one of the two possible conclusions can be drawn from the findings: (1) some methods generate more “noise” giving the illusion, especially with Eukaryota, that darkness relates more closely with disorder; (2) some methods reveal a hidden relationship between darkness and disorder that most predictors systematically fail to highlight. For now, and based on these predictors, we can state that darkness and disorder are two separate concepts where the latter is part of the former, but only a small part, as demonstrated in this study. Looking at the obtained results, one thing is evident—more research and the improvement of current tools of prediction are mandatory, as well as the development of new tools to explore the remaining secrets in the dark proteome.

## Figures and Tables

**Table 1:** Median values of darkness for dark and non-dark proteins from the data of 2014 [2] and 2016 [9].

Domain of Life	Darkness (2014)	Darkness (2016)
Archaea	4%	3.8%
Bacteria	4%	3.9%
Eukaryota	28%	26.9%
Viruses	65%	57.7%

**Table 2:** Median values of disorder for dark and non-dark proteins from the data of 2014 [2] and 2016 [9] for the MD predictor.

Domain of Life	Dark (2014)	Dark (2016)	Non-Dark (2014)	Non-Dark (2016)
Archaea	16%	22%	5%	5%
Bacteria	8%	12%	4%	4%
Eukaryota	40%	50%	13%	14%
Viruses	26%	30%	8%	10%

**Table 3:** Median values of disorder for dark and non-dark proteins from the data of 2014 [2] and 2016 [9] for the IUPred (2014) and IUPred2A (2016) predictors.

Domain of Life	Dark (2014)	Dark (2016)	Non-Dark (2014)	Non-Dark (2016)
Archaea	0%	11%	1%	20%
Bacteria	0%	16%	3%	23%
Eukaryota	10%	25%	6%	23%
Viruses	3%	18%	5%	22%

**Table 4:** Median values of disorder for dark and non-dark proteins from the data of 2016 [9] for the VSL2 predictor.

Domain of Life	Dark 2016	Non-Dark 2016
Archaea	11%	12%
Bacteria	12%	11%
Eukaryota	35%	19%
Viruses	18%	16%

**Table 5:** Standard deviation of disorder for dark and non-dark proteins from the data of 2016 [9] for the MD, IUPred2A and VSL2 predictors.

Domain of Life	MD	IUPred2A	VSL2
Archaea	0.301	0.018	0.137
Bacteria	0.293	0.114	0.141
Eukaryota	0.335	0.176	0.221
Viruses	0.314	0.158	0.187

## Data Availability

The datasets generated during and/or analyzed during the current study will be available in the Dark Proteome Database site [http://www.darkproteome.ws].
